# Fabrication of Aluminum Foam-Filled Thin-Wall Steel Tube by Friction Welding and Its Compression Properties

**DOI:** 10.3390/ma7096796

**Published:** 2014-09-19

**Authors:** Yoshihiko Hangai, Masaki Saito, Takao Utsunomiya, Soichiro Kitahara, Osamu Kuwazuru, Nobuhiro Yoshikawa

**Affiliations:** 1 Graduate School of Engineering, Gunma University, Kiryu 376-8515, Japan; E-Mail: t12801224@gunma-u.ac.jp; 2 SIT Research Laboratories, Shibaura Institute of Technology, Saitama 337-8570, Japan; E-Mail: utunomiy@sic.shibaura-it.ac.jp; 3 Hokudai Co., Ltd., Abira 059-1434, Japan; E-Mail: soichiro_kitahara@hokudai-jp.com; 4 Graduate School of Engineering, University of Fukui, Fukui 910-8507, Japan; E-Mail: kuwa@u-fukui.ac.jp; 5 Institute of Industrial Science, the University of Tokyo, Tokyo 153-8505, Japan; E-Mail: yoshi@telu.iis.u-tokyo.ac.jp

**Keywords:** cellular materials, composites, foam, friction welding, die casting, X-ray computed tomography (CT)

## Abstract

Aluminum foam has received considerable attention in various fields and is expected to be used as an engineering material owing to its high energy absorption properties and light weight. To improve the mechanical properties of aluminum foam, combining it with dense tubes, such as aluminum foam-filled tubes, was considered necessary. In this study, an aluminum foam-filled steel tube, which consisted of ADC12 aluminum foam and a thin-wall steel tube, was successfully fabricated by friction welding. It was shown that a diffusion bonding layer with a thickness of approximately 10 μm was formed, indicating that strong bonding between the aluminum foam and the steel tube was realized. By the X-ray computed tomography observation of pore structures, the fabrication of an aluminum foam-filled tube with almost uniform pore structures over the entire specimen was confirmed. In addition, it was confirmed that the aluminum foam-filled steel tube exhibited mechanical properties superior to those of the ADC12 aluminum foam and steel tube. This is considered to be attributed to the combination of the aluminum foam and steel tube, which particularly prevents the brittle fracture and collapse of the ADC12 foam by the steel tube, along with the strong metal bonding between the aluminum foam and the steel tube.

## 1. Introduction

Aluminum foam is expected to be used as a shock absorber of automobiles and a sound absorber of building materials owing to its high energy absorption properties, sound insulation properties and light weight [1,2]. Aluminum foam (Al foam) itself has low tensile and bending strengths. Therefore, Al foam is expected to be used as the core of composite materials by combining it with dense materials, such as an Al foam core sandwich panel [3,4] and an Al foam-filled tube [5,6,7,8,9,10], to improve its mechanical properties. In addition, it was demonstrated that the bonding between Al foam and dense materials further improves the performance of composites [7,10]. Typically, adhesives were used for bonding between previously fabricated Al foam and dense materials [2,7]. It is considered that the composites fabricated using an adhesive cannot be used under high-temperature conditions [3] and have limited usage owing to the difficulty in recycling [3] and considerable environmental concerns [11].

Clad bonding and friction stir welding (FSW) processes have been developed to overcome these problems using adhesives [3,4,12,13]. In these processes, a precursor foaming method [4,14] was employed. A foamable precursor, which is a mixture of Al and a blowing agent, was bonded to dense plates by clad bonding or FSW simultaneously with or after its fabrication, prior to the foaming of the precursor by heat treatment. These processes, which are adhesive-free, can achieve the metal bonding between Al foam and dense plates. However, clad bonding and FSW processes are limited to the fabrication of flat sandwich panels, and it is difficult to fabricate an Al foam-filled tube with metal bonding.

Recently, a friction surface coating process [15] has been developed by friction welding [16] for cylindrical hollow steel materials to easily coat the inner wall of the hollow with an aluminum alloy. In this process, bulk aluminum alloy is placed in the cylindrical hollow of the steel material, and a non-consumable cylindrical rotating tool is plunged into the hollow. Friction heat is generated between the bulk aluminum alloy and the rotating tool, which softens the aluminum alloy, causing it to flow plastically between the tool and the inner wall, and form a thin coating on the inner wall. During this process, it is expected that a metal bonding layer will be formed between the inner wall, and the aluminum alloy coating can be realized. It is expected that, if an Al foam precursor is placed alternatively to bulk aluminum alloy, the precursor can be easily bonded to the inner wall of the hollow of steel materials by friction welding. By the heat treatment of Al foam precursor-coated hollow steel materials, Al foam core steel composites can be obtained [17].

In this study, an Al foam-filled steel tube was fabricated by friction welding, which was expected to realize the metal bonding between Al foam and a thin-wall steel tube. First, an Al foam precursor was fabricated using the FSW route, in which blowing agent powder was mixed in Al plates by FSW [18,19]. The obtained precursor was placed in the steel tube; thereafter, the precursor was bonded to the steel tube by friction welding. By the heat treatment of the obtained precursor-coated steel tube, the foamed precursor filled the steel tube, forming an Al foam-filled steel tube. The diffusion bonding layer formed between the Al and the wall of the steel tube of the obtained precursor-coated steel tube, and the Al foam-filled steel tube were observed by scanning electron microscopy (SEM) to confirm whether metal bonding is achieved. The pore structures of the fabricated Al foam-filled steel tube were nondestructively observed by X-ray computed tomography (CT) and quantitatively evaluated to confirm whether uniform pore structures are formed over the entire samples. In addition, the compression properties of the obtained Al foam-filled steel tube were investigated in accordance with Japanese Industrial Standards JIS-H-7902 [20]. By comparing them with the compression properties of the Al foam and steel tube, the superior mechanical properties of the fabricated Al foam-filled steel tube were determined.

## 2. Results and Discussion

**Figure 1:**
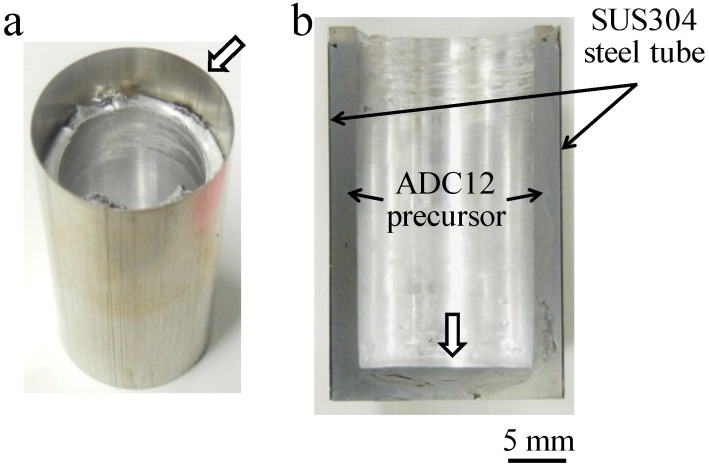
Fabricated precursor-coated steel tube: (**a**) Sample immediately after friction welding; (**b**) Cross-sectional image of (**a**).

Figure 1a,b show the fabricated precursor-coated steel tube and its cross section, respectively. Note that the top of the tube where the precursor was not coated and the bottom of the precursor indicated by void arrows, as shown in Figure 1a,b, were machined before the heating process. The coated precursor was found throughout the inner wall of the steel tube and no gap was observed, indicating that the precursor was attached firmly to the steel tube. Also, no deformation of the thin-wall steel tube was observed.

**Figure 2:**
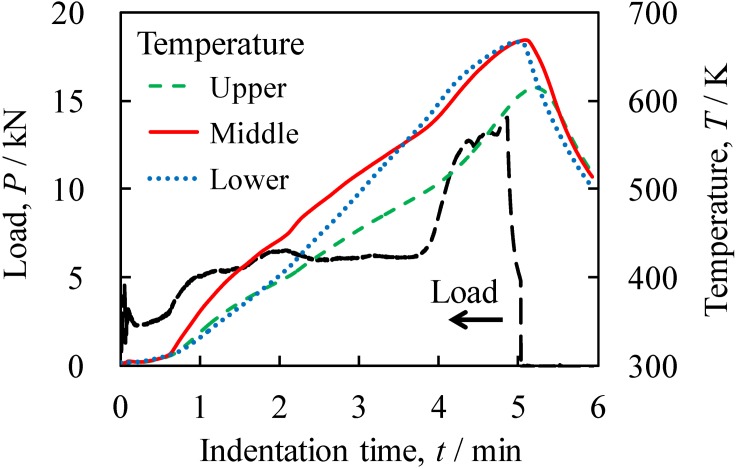
Variations in indentation load *P* and jig temperature *T* with respect to indentation time *t* during friction welding. Temperature was observed at the upper, middle and lower positions of the jig.

**Figure 3:**
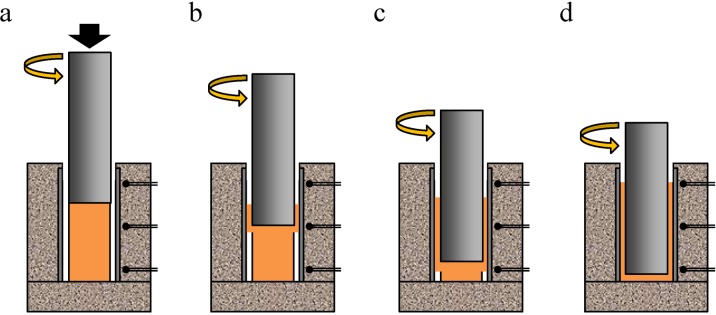
Schematic of deformation images of precursor during friction welding.

Figure 2 shows variations in indentation load *P* and jig temperature *T* with respect to indentation time *t* during friction welding. The temperature was observed at the upper, middle and lower positions of the jig. *t* is the time elapsed from the time when the tool first came into contact with the surface of the precursor. First, the indentation of the tool and the contact between the tool and the precursor, as shown in Figure 3a, caused a slight increase in load until approximately *t* = 0.5 min, although there was a shock load for a few seconds when the rotating tool touched the precursor. The obtained initial temperatures were almost the same regardless of the observation position. In this first stage, only the temperature of the precursor was increased by the friction between the precursor and the rotating tool, and the generated heat did not transfer rapidly to the jig because of the gap between the precursor and the steel tube. When the precursor was softened by the generated friction heat, the precursor deformed and touched the steel tube in the vicinity of the rotating tool, as shown in Figure 3b. The contact area between the rotating tool and the deformed precursor, and that between the deformed precursor and the steel tube increased as the indentation of the tool increased, increasing the deformation resistance of the precursor and the load at approximately *t* = 0.5–2.0 min. Simultaneously, the temperature of the middle position, which is in the vicinity of the rotating tool where the friction heat was generated, increased rapidly. The heat generated in the vicinity of the rotating tool that transferred into the fixing jig gradually increased the temperatures of the upper and lower positions. After approximately *t* = 2.0 min, as shown in Figure 3c, on the one hand, the increase in the deformation resistance of the precursor increased because of the increase in contact area. On the other hand, the deformation resistance of the precursor decreased owing to the increase in precursor temperature. The balance between the increase and decrease in deformation resistance caused the load to remain almost constant. In contrast, the temperature continuously increased, particularly at the lower position. As the tool indentation increased, the position of the tip of the rotating tool where the friction heat was mostly generated became closer to the lower position. Also, the friction between the side of the tool and the plastic flow of the Al foam precursor further increased the amount of heat. Finally, after approximately *t* = 4.0 min, as shown in Figure 3d, the gap between the precursor and the steel tube entirely disappeared. Therefore, the precursor below the tool that filled the gap between the rotating tool and the steel tube rapidly increased the load, owing to the enhancement of the plastic flow of the Al precursor between the tool and the steel tube. When the load reached a maximum value, the temperature also reached maximum values of approximately 670 K at the middle and lower positions and 620 K at the upper position. After *t* = 5.0 min, the tool was moved upward and unloaded, causing the temperature to decrease rapidly.

The temperature continuously increased during the indentation of the tool, because it is considered that the amount of heat released from the jig was lower than that of heat transferred to the jig. It was predicted that temperatures higher than that observed using the thermocouple, which was 2 mm away from the surface of the steel tube, would occur at the site of contact between the rotating tool and the precursor. However, as shown in Figure 1, foaming was not observed during friction welding.

**Figure 4:**
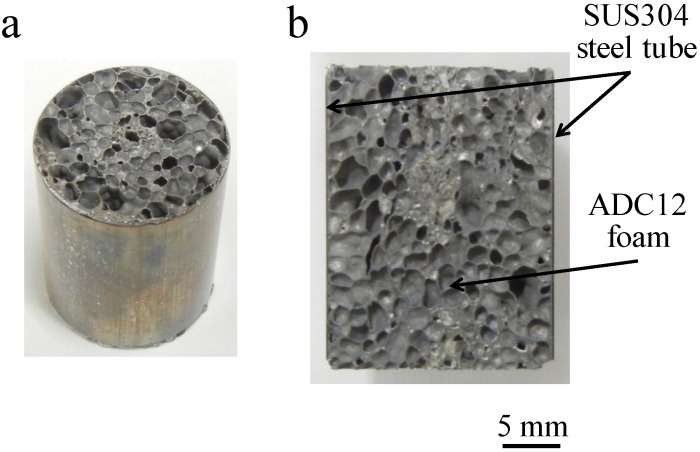
Fabricated Al foam-filled steel tube: (**a**) Compression test specimen; (**b**) Cross-sectional image of (**a**).

Figure 4a,b show the obtained Al foam-filled steel tube compression test specimen and its cross section, respectively. It can be seen that the Al foam completely filled the interior of the steel tube and that there were no locations showing insufficient foaming. No gap can be seen between the Al foam and the inner wall of the steel tube, indicating that the precursor remained in contact with the steel tube during heat treatment for foaming and that the Al foam attached firmly to the steel tube.

**Figure 5:**
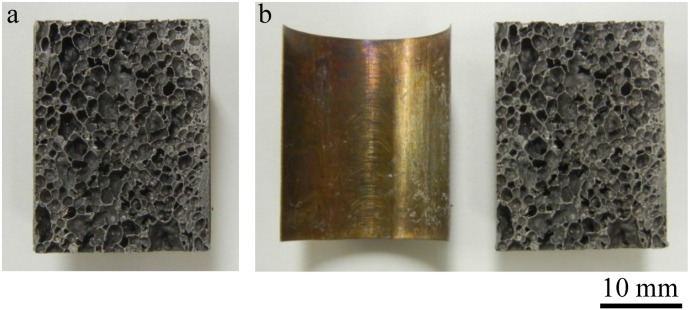
Fabricated Al foam-filled steel tube in which precursor was only placed in steel tube for foaming, *i.e.*, precursor was not bonded to steel tube by friction welding: (**a**) Cross-sectional image; and (**b**) Al foam removed from steel tube of (**a**).

Figure 5a shows an Al foam-filled steel tube similar to that shown in Figure 4, except that the precursor was not bonded to the tube by friction welding but only placed in the tube and foamed. The foaming temperature and time were set at 1003 K and 5.5 min, respectively. The foaming time for the precursor without friction welding was set longer than that for the precursor-coated steel tube because no foaming of the precursor can be observed at the foaming time of 4.5 min in this case. Although it appeared that the same Al foam-filled steel tube as shown in Figure 4 can be obtained, bonding between the Al foam and the steel tube was not realized, and the Al foam can be easily removed from the steel tube as shown in Figure 5b. Therefore, it is considered that the bonding of the precursor to the steel tube is necessary for fabricating the Al foam-filled tube, which realized sufficient bonding between the Al foam and the steel tube.

**Figure 6:**
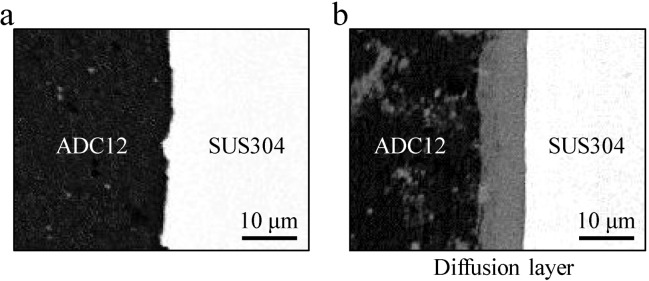
BEIs (backscattering electron images) of bonding interface of (**a**) precursor-coated steel tube and (**b**) Al foam-filled steel tube.

Figure 6a,b shows the typical backscattering electron images (BEIs) of the bonding interface, which was approximately at the middle height of the precursor-coated steel tube and the Al foam-filled steel tube, respectively. The black regions are ADC12 Al alloy, white regions are SUS304 steel, and the gray regions between ADC12 and SUS304 only observed in Figure 6b are presumed to be the diffusion bonding layers. It was expected that the diffusion bonding layer would be observed at the bonding interface of the precursor-coated steel tube, because friction heat was generated and a strong plastic flow of the precursor occurred continually for a few minutes, which resulted in the formation of a new surface and diffusion bonding between the precursor and the wall of the steel tube. However, as shown in Figure 6a, although no gap could be seen and the precursor and steel tube were closely attached, the diffusion bonding layer could not be observed for this magnification for the precursor-coated steel tube at the entire bonding interface. In contrast, as shown in Figure 6a,b diffusion bonding layer with a thickness in the order of 10 μm was formed at almost the entire bonding interface and bonding between the Al foam and the steel tube remained during the foaming process; the growth of the diffusion bonding layer appeared to be the result of extensive diffusion at the interface during the high-temperature foaming process. This tendency was consistent with the composite structural material with the ADC12 Al foam and the steel plate fabricated by FSW, which demonstrated that the diffusion bonding layer cannot be observed at the bonding interface of the ADC12 foam precursor and steel plate, but can be observed after heat treatment for the foaming of the ADC12 precursor [21]. However, this tendency was different from a previous study demonstrating that the diffusion bonding layer was formed at the bonding interface between an A1050 (pure Al) foam precursor and an SS400 steel block by friction welding [17]. This is considered to be due to the weak plastic flow of ADC12 Al alloy with Al–Si eutectic nature indicating brittleness [22,23]. Clearly, much more extensive studies are necessary to examine the formation of the diffusion bonding layer. Note that a small gray region with similar contrast with the diffusion bonding layer can be seen in the Al foam. This small gray region can also be observed in the precursor, as shown in Figure 6a; therefore, it is considered that the Fe–Al intermetallic compound (IMC) intrinsically contained in the ADC12 die-casting plates segregated during the foaming process. In addition, a scratched tiny steel tube contained in the precursor during friction welding due to the intense plastic flow may form the IMC by reaction with Al during the foaming process.

As described above in Figure 5, the Al foam-filled steel tube without friction welding indicated a weak bonding interface. Therefore, it was found that the attachment of the precursor to the steel tube by friction welding, which is considered to be mechanical bonding such as the anchor effect, is important for fabricating an Al foam-filled steel tube with the sufficient bonding of the Al foam and steel tube. Consequently, it was shown that an Al foam-filled thin-wall steel tube can be fabricated with strong metal bonding throughout almost the entire sample by friction welding.

It is a concern that the formation of the Fe–Al IMC layer may decrease the strength of the interface. It has been shown that the Al foam in Al foam/dense steel composite structures [24] and Al foam core sandwich panels with dense steel face sheets [12] have a lower strength than the bonding interface. Therefore, this result is not considered to be a problem, even if the Fe–Al IMC layer formed has a thickness in the order of 10 μm. Clearly, much more extensive studies are necessary to examine this assumption in more detail.

**Figure 7:**
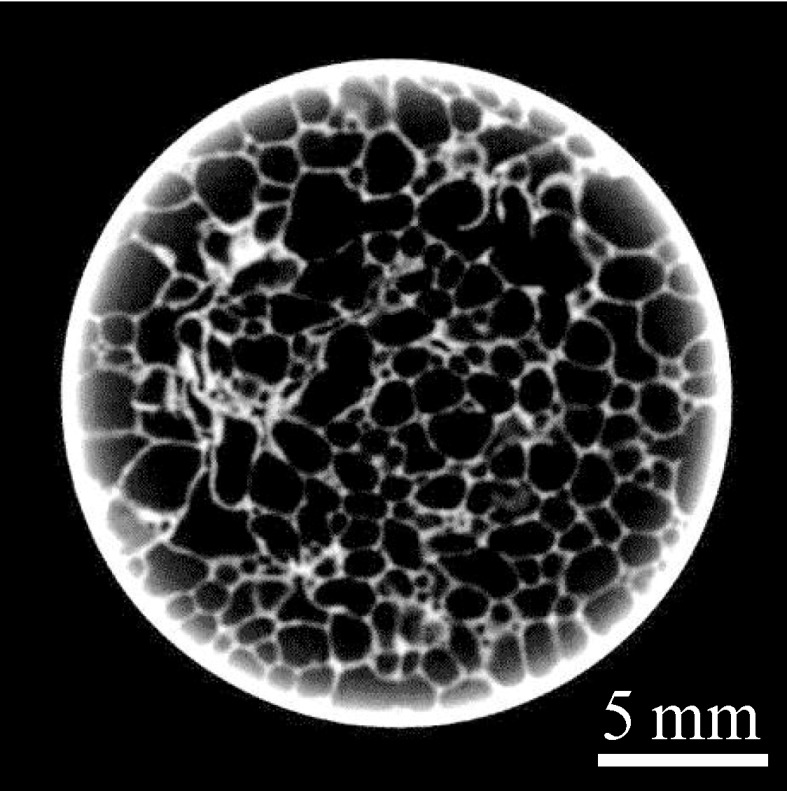
Typical cross-sectional X-ray CT image of Al foam-filled steel tube compression test specimen around the middle height of specimen.

Figure 7 shows a typical cross-sectional X-ray CT image of the Al foam-filled steel tube compression test specimen around the middle height of the specimen. The round white circle indicates the steel tube and the gray region in the white circle indicates the cell wall of the Al foam. It can be seen that almost uniform pore structures can be obtained.

**Figure 8:**
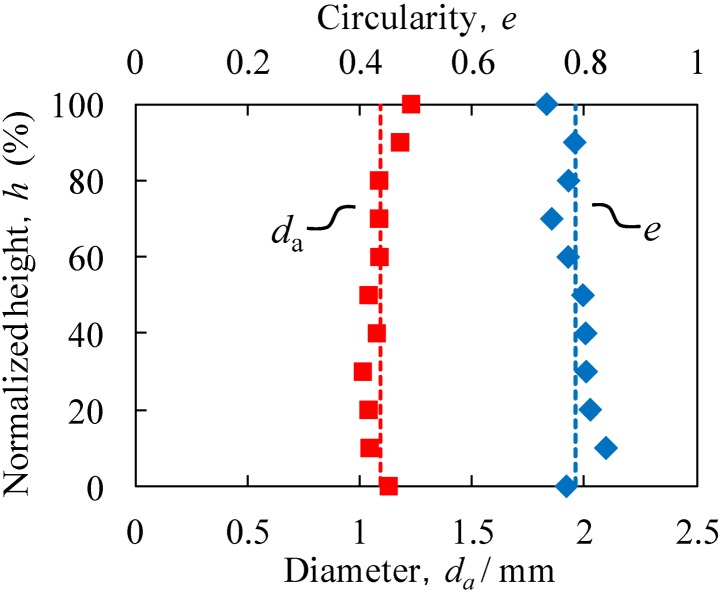
Relationship between specimen height and average diameter *d*_a_ and circularity *e* of pores.

Figure 8 shows the relationship between the location in the Al foam-filled steel tube (in the compression direction), expressed as the height *h* from the bottom of the specimen normalized by the specimen height, and the average diameter *d*_a_ and circularity *e* of the pores. It can be seen that the obtained Al foam-filled steel tube has almost uniform pore structures in the entire region. The average diameter and circularity of pores in the entire region were 1.09 mm and 0.78, respectively.

**Figure 9:**
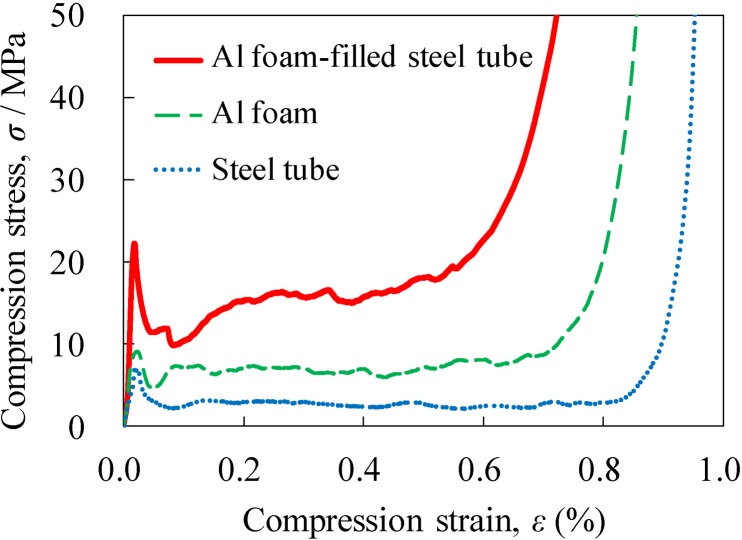
Stress–strain curves for fabricated Al foam-filled steel tube (*p* = 83.3%) along with those for Al foam (*p* = 85.9%) and steel tube.

**Figure 10:**
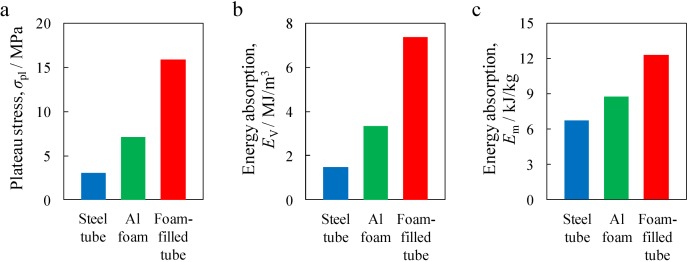
Comparison of mechanical properties between steel tube, Al foam and Al foam-filled steel tube: (**a**) Plateau stress; (**b**) Energy absorption per unit volume; and (**c**) Energy absorption per unit mass.

Figure 9 shows typical stress–strain curves under compression for the obtained Al foam-filled steel tube with porosity *p* = 83.3%, along with those for the Al foam with *p* = 85.9% and the steel tube. The steel tube alone was heat-treated similarly to the Al foam-filled steel tube during the foaming process. Three specific regions, namely, the elastic region at the initial stage that indicated the initial peak stress, the plateau region with a nearly constant stress up to a large strain and the densification region where the stress increased markedly, can be clearly observed in all the stress–strain curves, which were similar to those for the conventional Al foam [1,25]. The compression stress of all the samples decreased after attaining the initial peak stress, and thereafter, the compression stress remained almost constant, but slight fluctuation, which was similar to that of the Al foam based on a brittle alloy [26,27,28]. Figure 10 shows the plateau stress σ_pl_, energy absorption per unit volume, *E*_V_, and energy absorption per unit mass, *E*_m_, values of the steel tube, Al foam and Al foam-filled steel tube. It can be seen that the Al foam-filled steel tube exhibited mechanical properties superior to those of the Al foam and steel tube, which is considered to be attributed to the combination of the Al foam and steel tube, in addition to the realization of good bonding between the Al foam and the steel tube.

**Figure 11:**
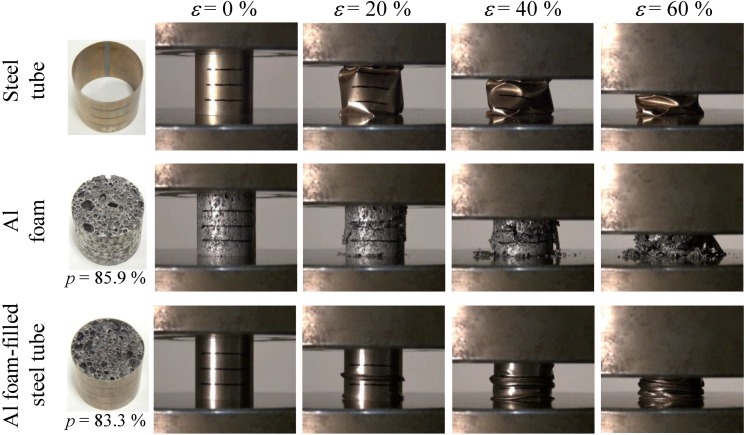
Deformation behaviors of steel tube, Al foam and Al foam-filled steel tube.

Figure 11 shows deformation images for different strains under compression loading, which correspond to the stress–strain curves shown in Figure 9, for the steel tube, Al foam and Al foam-filled steel tube. The steel tube alone exhibited unstable buckling that resulted in fluctuation in the stress–strain curve but indicated an almost constant stress. The ADC12 Al foam alone exhibited localized brittle fracture and fragments of the collapsed foam were observed. This brittle fracture results in some fluctuation in the stress–strain curve, as observed in the steel tube alone. The Al foam-filled steel tube exhibited repetition of buckling, which is different from the deformation behaviors of the steel tube and Al foam, resulting in a marked fluctuation in the stress–strain curve. Note that the localized brittle fracture and fragments of the collapsed ADC12 Al foam alone could not be observed, which was attributed to the existence of the steel tube. Consequently, it is considered that a steel tube with metal bonding to Al foam is efficient for preventing the collapse of a brittle Al foam and improving the mechanical properties of Al foam.

## 3. Experimental Procedure

### 3.1. Materials

**Figure 12:**
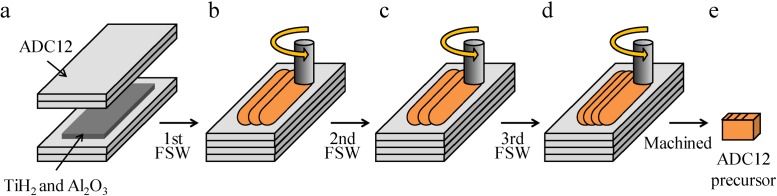
Schematic illustration of fabrication process of ADC12 Al foam precursor using FSW route.

Figure 12 shows a schematic illustration of the fabrication process of the Al foam precursor. As the starting material, Al–Si–Cu aluminum alloy ADC12 (equivalent to A383.0 aluminum alloy) high-pressure die-casting plates of 3 mm thickness were used. First, as shown in Figure 12a, four die casting plates were stacked with TiH_2_ powder (<45 μm, 1 mass %) as the blowing agent and Al_2_O_3_ powder (~1 μm, 5 mass %) as the stabilization agent were distributed between the second and third plates. Next, as shown in Figure 12b–d, FSW was conducted for the laminated plates to mix the powders in the ADC12 plates and to join the laminated plates. FSW was carried out using an FSW machine (Hitachi Setsubi Engineering Co., Ltd., Tokyo, Japan). The FSW tool was cylindrical and had a screw probe. The diameter of the tool shoulder was 19.5 mm, and the diameter of the tool probe was tapered from 8 to 4 mm and its length was 9.3 mm. The tool rotation speed was 1000 rpm and the welding speed was 50 mm/min. A tilt angle of 3° was used. The multipass FSW technique [29], as shown in Figure 12b–d, was applied for the FSW region to be stirred three times to obtain a large amount of precursor and to mix the powders in the Al plates thoroughly. Three passes were conducted for the first and second FSWs, and four passes were conducted for the third FSW. The precursor of 9.5 × 17 × 30 mm was machined from the region subjected to FSW.

**Figure 13:**
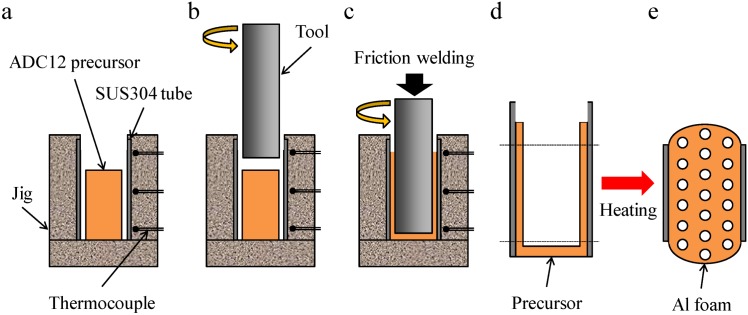
Schematic illustration of fabrication process of ADC12 Al foam-filled thin-wall SUS304 steel tube by friction welding.

Figure 13 shows a schematic illustration of the fabrication process of the Al foam-filled steel tube. First, as shown in Figure 13a, an SUS304 steel tube 0.1 mm in wall thickness, 20 mm in diameter and 40 mm in height was set in the fixing jig. The surface of the inner wall of the steel tube was roughened with a #320 SiC paper to improve the bondability with the Al. Then, the obtained precursor was set in the steel tube. Next, as shown in Figure 13b, a rotating tool of SUS304 steel 15 mm in diameter was inserted from above into the steel tube at a feed rate of 5 mm/min and a rotational speed of 800 rpm. The tool was inserted until its tip was 3 mm above the bottom of the steel tube. Friction heat was generated between the precursor and the rotating tool during friction welding, and the plastic flow of the softened precursor induced the filling of the volume between the tool and the steel tube, as shown in Figure 13c. The temperature during friction welding was measured using a thermocouple placed at the upper, middle and lower positions of the fixing jig located 2 mm from the surface of the steel tube. Finally, the obtained precursor-coated steel tube, whose upper and lower parts were machined to a height of 25 mm as shown in Figure 13d, was placed in a preheated electric furnace held at 1003 K, resulting in the fabrication of the Al foam-filled steel tube as shown in Figure 13e. The holding time in the furnace (foaming time) was set at 4.5 min. The foamed samples of the upper and lower parts were machined by electrodischarge machining, and an Al foam-filled steel tube compression test specimen with dimensions of *ϕ* = 20 × 20 mm was obtained.

### 3.2. Observation of Microstructures

An EPMA-1610 electron probe microanalyzer (Shimadzu Corporation, Kyoto, Japan) was employed to observe BEIs of the diffusion bonding layer formed between the Al and the steel tube of the obtained precursor-coated steel tube, and Al foam-filled steel tube. The observation surface was ground using SiC papers (up to #2400), followed by alumina (1 μm) polishing before the BEI observation.

### 3.3. Evaluation of Pore Structures

The porosity *p* (%) of the compression test specimen was evaluated as:

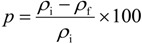
(1)
where *ρ*_i_ is the density of the precursor without a steel tube before heating and *ρ*_f_ is the density of the Al foam part of the compression test specimen. The density of ADC12 Al alloy [30] is used for *ρ*_i_. *ρ*_f_ was evaluated as follows. First, the mass of the compression test specimen with a steel tube, *m*_AFT_, was measured. Next, by multiplying the density of the steel tube, which was previously evaluated by Archimedes’ principle, and the volume of the steel tube of the compression test specimen, the mass of the steel tube, *m*_tube_, was estimated. Finally, *ρ*_f_ was evaluated as:

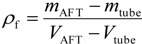
(2)
where *V*_AFT_ is the volume of the compression test specimen and *V*_tube_ is the volume of the steel tube of the compression test specimen.

To evaluate the size and circularity of the pores of the Al foam, X-ray CT observations were performed on the compression test specimen using an SMX-225CT microfocus X-ray CT system (Shimadzu Corporation, Kyoto, Japan). The X-ray source was tungsten. A cone-type CT system, which produces three-dimensional images, was employed. In this system, only one rotation of the specimen was sufficient to obtain a three-dimensional volume image, which consisted of a set of CT images with a slice pitch equal to the length of one pixel in each CT image. The resolution of each CT image was 512 × 512, and the pixel length was 51 μm. The X-ray tube voltage and current were 80 kV and 70 μA, respectively.

The average diameter *d*_a_ and circularity *e* distributions of the pores were evaluated from two-dimensional cross-sectional X-ray CT images of the Al foam using WinROOF image processing software (Mitani Corporation, Fukui, Japan). An appropriate threshold was set to distinguish the cell walls and the pores, and binarized X-ray CT images were established for the evaluation. Pores with areas of less than 0.3 mm^2^ were excluded in the evaluation owing to the resolution of the X-ray CT images [31]. The circularity *e* of the pores was evaluated as:

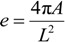
(3)
where *A* is the pore area and *L* is the pore perimeter. A value of circularity closer to 1 indicates a more circular pore.

### 3.4. Compression Tests

Compression tests were carried out at room temperature in ambient air using an Autograph AG-100kNG universal testing machine (Shimadzu Corporation) at a strain rate of 3.3 × 10^−3^·s^−1^. At the same time, the compression deformation of the specimen was recorded using a digital video camera.

From the obtained stress–strain curves, the plateau stress *σ*_pl_ was defined as the average stress for a strain of 20%–30% [20]. The energy absorption per unit volume, *E*_V_, was defined as the area under the stress–strain curve up to 50% strain as in [20]:

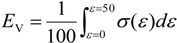
(4)


The energy absorption property per unit mass, *E*_m_, was evaluated by multiplying the volume of the sample by *E*_V_, then dividing by the mass of the sample. Note that the steel tube was assumed to be solid when calculating the cross-sectional area and volume for the evaluation of the plateau stress and energy absorption.

## 4. Conclusions

In this study, an Al foam-filled steel tube, which consisted of ADC12 Al foam and a thin-wall steel tube, was successfully fabricated by friction welding. The following conclusions were obtained by experimental investigations.

(1) A foamable precursor can be coated on the inner wall of the steel tube with strong bonding by friction welding.
(2) By the heat treatment of a precursor-coated steel tube, an Al foam-filled steel tube can be obtained. A diffusion bonding layer with a thickness of approximately 10 μm was formed, indicating strong bonding between the Al foam and the steel tube.
(3) By the X-ray CT observation of pore structures, an Al foam-filled tube with almost uniform pore structures over the entire specimen can be realized.
(4) The Al foam-filled steel tube exhibited mechanical properties superior to those of the Al foam and steel tube. This is considered to be attributed to the combination of the Al foam and steel tube, which particularly prevents the brittle fracture and collapse of the ADC12 foam by the steel tube, along with the strong metal bonding between the Al foam and the steel tube.
